# Global Impacts of Marine Methanethiol Emissions and Chemistry in the Atmosphere

**DOI:** 10.1021/acs.est.5c02019

**Published:** 2025-09-18

**Authors:** Linia Tashmim, William C. Porter, Timothy H. Bertram, Delaney B. Kilgour, Andrew Rollins

**Affiliations:** 1 Department of Environmental Sciences, University of California, Riverside, California 92521, United States; 2 Department of Chemistry, 5228 University of Wisconsin-Madison, Madison, Wisconsin 53706, United States; 3 Chemical Sciences Division, NOAA Earth System Research Laboratory, Boulder, Colorado 80305, United States

**Keywords:** oxidation, sulfur, aerosol, GEOS-Chem, fluxes, dimethyl sulfide (DMS), methanethiol (MeSH), methanesulfonic acid (MSA)

## Abstract

Oceanic emissions of dimethyl sulfide (DMS) have long been known to influence aerosol particle composition, cloud condensation nuclei (CCN) concentration, and Earth’s radiative budget. However, the impact of oceanic emissions of methanethiol (MeSH), a sulfur compound produced by the same oceanic precursor as DMS, has been relatively less explored. The gas-phase oxidation of MeSH has a higher effective yield of SO_2_ and a shorter oxidative lifetime compared to DMS, highlighting the relevance of this pathway for the modeled representation of particle formation, growth, and CCN abundance in the marine atmosphere. Here, we use the global chemical transport model GEOS-Chem to explore possible scenarios representative of specific environmental conditions and MeSH emission schemes based on previous experimental studies. We further implement and test previously reported chemical mechanisms for MeSH oxidation, along with additional improvements, highlighting key uncertainties and sensitivities for regional and global sulfur budgets. We place our results in the context of recent modeling updates to DMS chemistry and cloud processing, which further impact SO_2_ production in the marine atmosphere in parallel with MeSH oxidation. Within the overall marine sulfur budget, our findings highlight that MeSH plays a significant role in SO_2_ production in the marine atmosphere, contributing to regional surface layer concentration increases of up to 40–60%. These results point to the importance of MeSH for efforts aimed at improving the modeled representation of sulfur spatiotemporal patterns relevant to air quality predictions and climate impact assessments.

## Graphical abstract

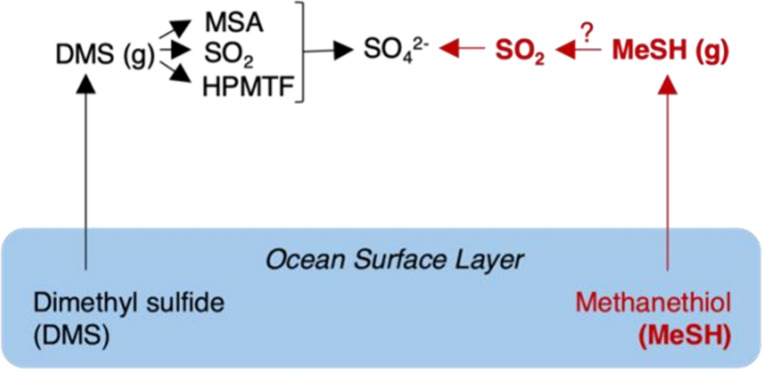

## 1 Introduction

Emissions of sulfur species from marine sources play a substantial role in Earth’s overall radiative budget and are considered a major source of uncertainty in predicting air quality and climate change.^1–4^ DMS emissions from the ocean stand out as the primary source of sulfur to the marine atmosphere, particularly over remote regions where the influence from anthropogenic sources of sulfur is relatively minor.^5–8^ Global sea-to-air DMS fluxes were estimated to fall within the range of 17.6–35 Tg S yr^–1^ based on the Lana DMS climatology,^9^ with more recent updates estimating an annual value of 27.1 Tg S yr^–1^.^10^ Alongside volcanic sulfur emissions, this marine biogenic source is believed to have been a dominant contributor to preindustrial atmospheric sulfate aerosol.^11^

Despite the long-recognized role of DMS chemistry in the marine atmosphere, knowledge regarding intermediate products of DMS oxidation and the kinetics involved in their oxidation processes is still developing, resulting in a wide range of estimates for final sulfur dioxide (SO_2_) yields (31–98%).^12–17^ Estimating the total budget of SO_2_ is important for many atmospheric questions as it is a precursor of sulfuric acid (H_2_SO_4_) and non-sea-salt sulfate aerosols (nssSO_4_^2–^), key players in Earth’s overall radiative budget.^14,18^ Earlier efforts to determine the overall SO_2_ budget in the marine boundary layer (MBL) depended on the assumption of nearly 100% SO_2_ yield from DMS oxidation.^18^ However, this high yield of SO_2_ from DMS oxidation appears unrealistic considering later evidence for the formation of oxidation products such as methanesulfonic acid (MSA) and hydroperoxymethyl thioformate (HPMTF) through laboratory and observational studies.^16,19,20^ Recent experimental and observational evidence shows that irreversible loss of DMS-derived stable intermediate HPMTF to clouds within the marine boundary layer acts as the major loss process for HPMTF, reducing SO_2_ production from DMS by 35% overall when this mechanism is included in a global model.^19^ These previously overlooked loss processes for sulfur derived from DMS contribute to an even greater underestimation of SO_2_ in comparison to airborne observations. This points to the possibility of unrepresented marine sulfur species necessary for the closure of the marine SO_2_ budget, as suggested in other recent works.^21–25^

MeSH is one such volatile sulfur species produced from the same oceanic precursor as DMS named dimethylsulfoniopropionate (DMSP).^24,26–28^ Since the accurate estimation of SO_2_ is crucial in calculating the global natural aerosol burden and associated aerosol indirect radiative forcing, inclusion of missing SO_2_ sources such as MeSH and relevant chemical processes could be an important step toward addressing this uncertainty in air quality models and global atmospheric chemistry models.^21,23–25,29,30^ Recent work shows lower modeled SO_2_ volume mixing ratios compared to the observations for most altitudes and latitude bins in remote regions over the Pacific, Atlantic, and Southern Oceans using global aerosol models.^29^ In addition to changes in emissions inventories and chemical mechanisms, observationally constrained modeling approaches involving ATom aircraft campaigns have also contributed to improved representation of SO_2_ trends.^31^ Previous work also found a general underestimation of observed SO_2_ when evaluating expanded DMS oxidation chemistry in the GEOS-Chem chemical transport model.^17^ In earlier research, it was found that a major portion of marine biogenic sulfur cycling passes through the MeSH pathway rather than DMS.^27^ Other more recent work has shown that inclusion of MeSH emissions in a global chemistry-climate model leads to a 30–70% increase in the sulfate aerosol burden over the Southern Ocean.^32^

Here, we evaluate the potential global impacts of MeSH emissions and oxidation using GEOSChem, integrating previously developed DMS oxidation chemistry with additional MeSH oxidation reactions. As part of this work, we add hypothetical sea-to-air flux emissions of MeSH based on existing DMS emissions climatologies scaled by DMS:MeSH emission ratios estimated from recent observations^24^ and explained in greater detail in the Supporting Information. We further evaluate the atmospheric impacts of these modeled MeSH emissions by quantifying their contribution to SO_2_ formation, identifying uncertainties in the chemical mechanism, and discussing improvements necessary to better represent the impacts of MeSH on the atmospheric chemistry and climate. We hypothesize that the inclusion of MeSH chemistry will increase H_2_SO_4_ production overall, with implications for new particle formation and growth and CCN abundance in the atmosphere. Better understanding of the relative importance of MeSH oxidation intermediate products in contrast with DMS will give better estimates of modeling of sulfur compounds in the marine atmosphere, thereby improving the modeled predictions of air quality and climate.

## 2 Methodology

We use the GEOS-Chem global chemical transport model v12.9.3 to simulate MeSH chemistry and oxidation products (https://github.com/geoschem/geos-chem/tree/12.9.3). The GEOS-Chem model includes a detailed mechanism for oxidant-aerosol chemistry in the troposphere and stratosphere with kinetics and products in the standard GEOS-Chem chemical mechanism generally following JPL and IUPAC recommendations.^33–38^ DMS emission fluxes from the ocean are controlled by a linear gas transfer velocity parametrization, which is dependent on sea surface temperature and wind speed^39^ coupled with a climatology of concentrations in seawater.^9^ Our added MeSH emission fluxes from the ocean start with the same DMS seawater climatologies, with the parametrization adjusted based on MeSH molecular properties and further divided by a scaling factor of 5 based on previously measured flux ratios.^24^ We performed several simulations and sensitivity cases to assess the MeSH impacts. To represent a baseline without MeSH impacts, we use a previously expanded DMS chemical mechanism^17^ merged with the CH_3_SO_*x*_ chemistry from Table S1 (except the first four reactions involving MeSH), which we refer to as “BASE”. We further implement and evaluate a custom chemical mechanism for MeSH oxidation, referred to hereafter as “MOD”, representing an integration of a previously explored MeSH oxidation mechanism with further updates to include additional reactions of known intermediates according to the Master Chemical Mechanism (MCM) v3.3.1 on top of our extended DMS oxidation mechanism.^17,24^ More detailed information on sensitivity cases, reaction choices, and other model details is discussed in the Supporting Information, including a list of reactions considered for MeSH oxidation in Table S1.

## 3 Results and Discussion

### 3.1 MeSH Burden and Oxidation Pathways

In the MOD case, surface MeSH mixing ratios (Figure 1b) are highest in the North Pacific and North Atlantic Oceans for the Northern Hemisphere (NH) and in certain regions of the Antarctic Circle in the Southern Hemisphere, reflecting the seasonality and spatial distribution of the underlying DMS climatologies in the model as seen in our previous work.^17^ While the DMS climatology appears to be a key factor influencing the peak emission patterns, the relative contribution of chemical loss pathways in each hemisphere also plays an important role in shaping the spatial distribution of emitted sulfur species and their oxidation products.^17,40^

**Figure 1:**
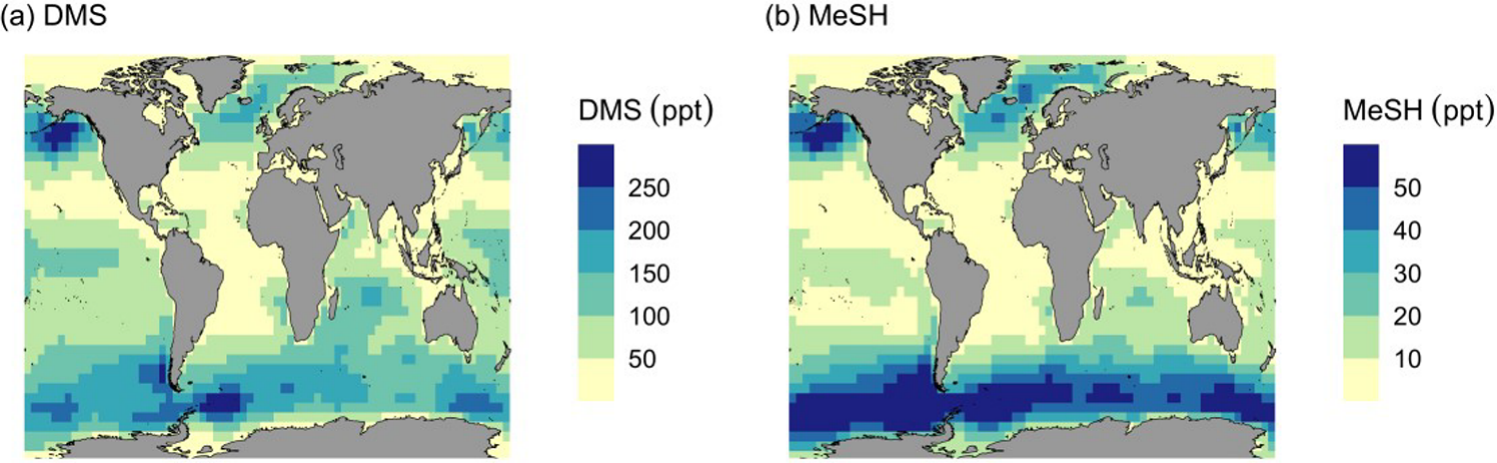
Geographic distribution of annual mean surface for (a) DMS and (b) MeSH mixing ratio (ppt) for 2018 with simulation MOD.

We find that the global burden of MeSH and DMS in the MOD simulation is 11.7 Gg S and 58.0 Gg S, respectively, with a global annual average lifetime of 4.1 h for MeSH. We also calculate a global annual DMS emission of 21.5 Tg S yr^–1^, which falls within previously reported ranges (17.6–35 Tg S yr^–1^) based on the same DMS climatology,^9^ with a corresponding global annual MeSH emission of 4.2 Tg S yr^–1^. Other recent work has reported global annual MeSH emissions of 5.7 ± 0.6 Tg S yr^–1^ based on an updated and further developed MeSH emission scheme.^32^ The mean surface global annual mixing ratio was found to be 15.2 ppt for MeSH over the surface of the oceans, slightly lower than reported by recent measurements.^24,41^ This value also falls within the range of atmospheric MeSH mixing ratios (10–55 ppt) measured across a broad latitudinal band (37° S to 67° S) in the Southern Hemisphere.^42^ Factors that we expect to influence surface concentrations include uncertainty in emissions and transport, oxidant concentrations, and MeSH chemical loss rates. Additional discussion on modeled emission fluxes of DMS and MeSH, as well as associated uncertainties, is available in the Supporting Information.

As shown in Figure 2a, the MOD simulation results in widespread SO_2_ enhancements relative to BASE, with an overall 12.1% increase in the global annual mean surface layer SO_2_ mixing ratio and local increases reaching nearly 40–60% in the Eastern Tropical Pacific, South Atlantic, and Indian Oceans. These changes indicate that the inclusion of gas-phase MeSH oxidation reactions results in a higher net concentration of gas-phase SO_2_ relative to that of the BASE simulation. This increased SO_2_ concentration is likely due to the shorter global annual average lifetime of MeSH (4.1 h) relative to DMS (21.6 h) and faster chemical loss to SO_2_, consistent with previous findings.^24^ With this shorter lifetime, an efficient chemical conversion of MeSH oxidation derived sulfur containing intermediates, such as CH_3_SO, CH_3_SOO, and CH_3_SO_2_, to SO_2_ results in an increase in SO_2_ formation in regions where these reactions occur at their most favorable rates. Note that these three SO_2_ precursors are also part of the previously developed DMS oxidation mechanism and are included in the DMS oxidation mechanism used here in the BASE simulation. To further quantify impacts on the overall sulfur budget, we also calculate SO_2_ yield, defined here as the global SO_2_ burden relative to total sulfur emissions. Based on this metric, we find that including CH_3_SH emissions and oxidation chemistry results in a 2.8% increase in global annual average yield. This supports the hypothesis that CH_3_SH contributes to a more efficient conversion of total sulfur emissions to SO_2_.

**Figure 2:**
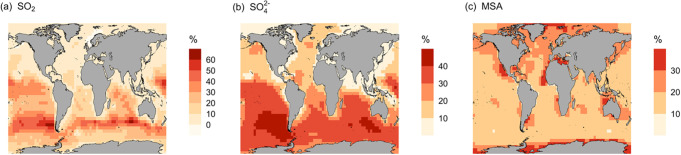
Geographic distribution of percent change in the simulated annual mean surface layer (a) SO_2_, (b) SO_4_^2–^, and (c) MSA for simulation MOD relative to BASE for the year of 2018. The geographic distributions of absolute change in simulated annual mean surface layer sulfate aerosol mass concentration (μg m^–3^) for simulation (d) MOD and (e) difference between simulations from its baseline, Δ = MOD – BASE for the year of 2018 are shown in the lower panel.

This increase in SO_2_ is expected to enhance the availability of gas-phase H_2_SO_4_ for new particle formation by nucleation, slightly counterbalancing the reduction in SO_2_ concentrations previously observed with the expansion of the simplified DMS oxidation scheme in GEOS-Chem.^13,16,17,19,43^ Figure 2b also shows an increase in global annual mean surface layer total SO_4_^2–^ over the ocean of about 19.1% with regional increases reaching up to 30–50% for the Southern Atlantic Ocean, Southern Ocean, and part of the Southern Indian Ocean. This increase could be the result of several underlying drivers: (1) MeSH oxidation chemistry is increasing the SO_2_ concentration, which further oxidizes to sulfate mostly via in-cloud oxidation with H_2_O_2_, (2) reduced gas-phase sulfur species such as CH_3_SO_3_ also contribute to sulfate formation in our mechanism following other works,^13,17,44^ and/or (3) the mechanism we implement here for MeSH oxidation does include additional pathways for the formation of one of the important sulfur species, MSA, via oxidation of CH_3_SO_2_OO with HO_2_ and CH_3_O_2_.^25^ The relative importance of these drivers on SO_2_ and MSA increases varies regionally, while enhanced SO_4_^2–^ concentrations are caused primarily by CH_3_SO_3_ oxidation chemistry. These additional reactions are responsible for a general increase in the global annual mean surface layer MSA over the ocean by about 22.1% (Figure 2c). Local increases reach up to 20–40% for the Southern Ocean near Antarctica and roughly 20–30% for the North Atlantic and North Pacific Oceans, consistent with the regions of high biological productivity and DMS emissions (and therefore, in our simulations, also MeSH emissions). While some of this MSA deposits back to the surface, about 86% of that further oxidizes to sulfate, thereby contributing to surface sulfate increases. While there are major uncertainties in this modeling effort, especially due to limited knowledge about the global consistency of assumed DMS:MeSH emission ratios as well as limited direct experimental evidence for many of the oxidation pathways involving MeSH intermediates, the MeSH oxidation mechanism used here provides meaningful information on their expected potential contributions to the marine atmospheric sulfate budget using current assumptions.

We also analyze the impact of this proposed MeSH oxidation chemistry on sulfate aerosol mass concentration, as shown in Figure 2d,e. We find an increase of around 19.1% for global annual mean surface layer sulfate aerosol mass concentrations (Figure 2e), which could be attributed to an increase in the precursor concentration. The additional formation of SO_2_, MSA, and more efficient CH_3_SO_3_ chemistry in the presence of MeSH emission in the MOD simulation collectively acts as the underlying reason for this increasing trend of sulfate aerosol mass concentrations. This increasing trend in sulfate aerosol mass could have important implication for air quality on the regional scale.

### 3.2 Comparison with Observations

Unlike the recent work,^32^ which relies on global MeSH emissions derived from a compilation of seawater data sets, our study not only examines the spatial distribution of MeSH and its oxidation products but also directly compares modeled atmospheric MeSH with observations. This approach provides additional insights for improving emission estimates and enhancing the model performance. Details of the observational comparisons are discussed in the following sections.

#### 3.2.1 Comparison with Ocean Surface Observations

We evaluate model output through a comparison with measurements of DMS and MeSH from the Aerosol Growth in the Eastern North Atlantic (AGENA) campaign, which sampled at the Eastern North Atlantic site on Graciosa Island, in the Azores (39.0916°N, 28.0257°W) from June 1, 2022, to July 15, 2022, using a Vocus PTRToFMS, which saved the measurements at 1 Hz.^45^ The hourly averaged concentration of DMS and MeSH for each day of measurement is shown in Figure 3, and the location of measurement is shown in Figure S3. For this comparison, the model is sampled at the time and location of the AGENA campaign, and an hourly averaged output was used to compare with the observations, which were also averaged by hour.

**Figure 3:**
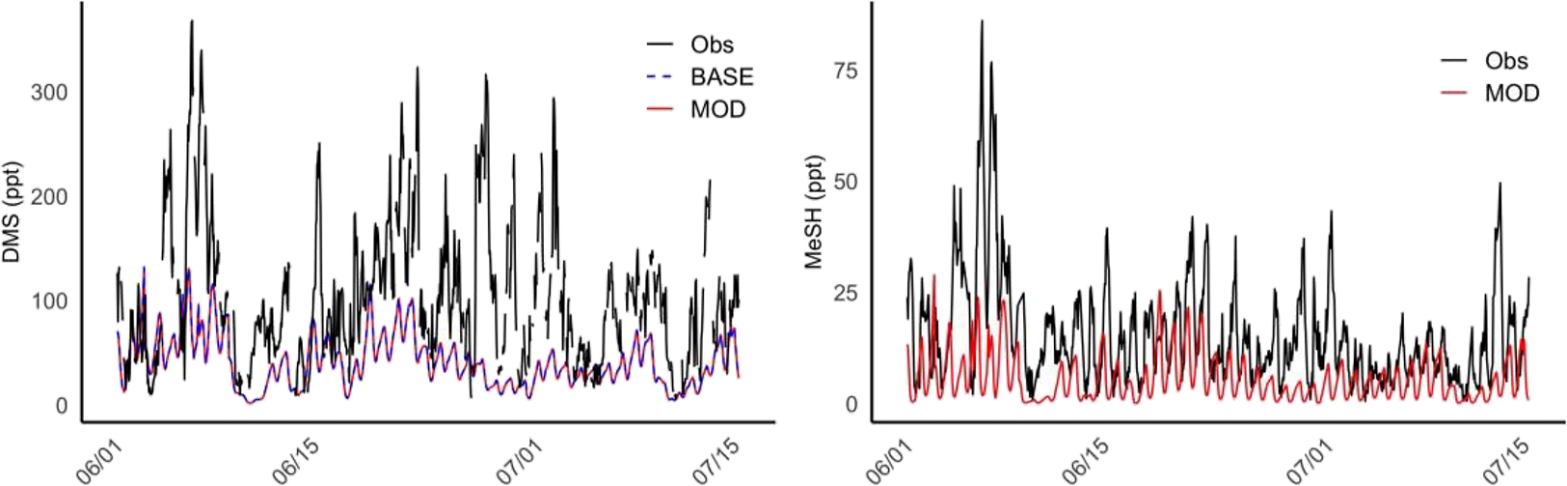
Time series of DMS (left) and MeSH (right) from June 1, 2022, to July 15, 2022. The black, blue dashed, and red lines correspond to observation (Obs) and modeled concentration without (BASE) and with (MOD) MeSH oxidation chemistry, respectively. The specific locations of measurement for DMS and MeSH are shown in Figure S3.

In general, modeled DMS concentrations are significantly lower than those observed during AGENA for most of the days of measurements. Maximum observed concentrations reach approximately 385 ppt DMS, while modeled concentrations do not exceed 131 ppt (Figure 3). Based on the similarities between the BASE and MOD cases, we conclude that modeled DMS concentrations are largely unaffected by the addition of MeSH oxidation chemistry. For MeSH, the model captures some aspects of the temporal variability in comparison to observations, although it clearly underpredicts the magnitude and fails to reproduce several short-lived episodic enhancements (e.g., early June). Days with high observed DMS concentrations often coincide with elevated MeSH, suggesting coemission; however, the modeled underprediction of both species points to uncertainties in the underlying DMS emission inventories and the highly simplified treatment of MeSH emissions. Additional uncertainties may arise from the atmospheric chemistry representation and the limited observational constraints on MeSH.

We also performed a correlation analysis for hourly averaged observed and modeled DMS and MeSH mixing ratios, colored by the hour of day (local time, UTC-0) as shown in Figure 4. For the observation shown in Figure 4a, data points show a wider spread, particularly in the higher range of DMS concentrations, again demonstrating the trend toward underprediction in modeled values. Observed data show a strong diurnal cycle for MeSH, with lower concentrations associated with afternoon hours and which is reported earlier.^45^ Model results differ, with a tighter correlation and hourly bands apparent in the DMS:MeSH scatter plot, suggesting that the modeled connection between the two species may be overly simplistic and tied to unrepresentative diurnal emissions patterns. The higher *R* ^2^ value (0.77) in the model output in Figure 4b suggests a closer correlation between the two species than is apparent in the observed data, pointing to the possibility of missing emission variability or other confounding factors. Beyond emissions, model agreement is affected by the differing chemical lifetimes of these species during the day. The longer lifetime of DMS compared to MeSH can further complicate correlations, as high concentrations from 1 day can potentially influence later days as well. By subsetting the hourly averaged data for nighttime (22 – 04 UTC) when daytime chemistry is inactive, we find that the *R* ^2^ significantly improves for the modeled output, even though the slope remains the same (Figure S4).

**Figure 4:**
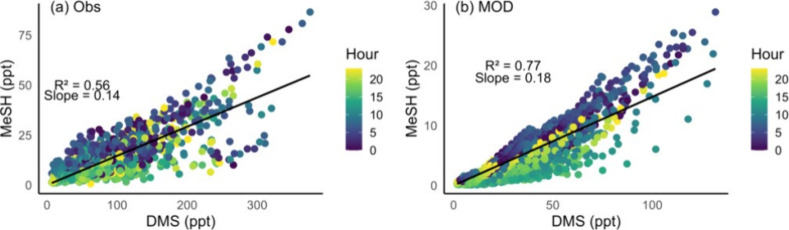
Correlation of DMS and MeSH mixing ratios at hourly averaged time colored by hour of day of the measurement for AGENA observations (a) and modeled concentration with simulation MOD sampled at the time and location of the AGENA campaign (b). Hour of the day is in local time (UTC – 0). The linear least-squares best fit is plotted as a solid black line. Location and period of measurement are the same as used in Figure 3.

The observed and measured diel profiles of DMS and MeSH are shown in Figure 5. In both cases, we find a general modeled underprediction, though general patterns agree between model and observations. Notably, model estimates agree relatively well with the lowest range of diurnal observations, presumably representing those days characterized by reduced upwind emissions or stronger chemical losses.

**Figure 5:**
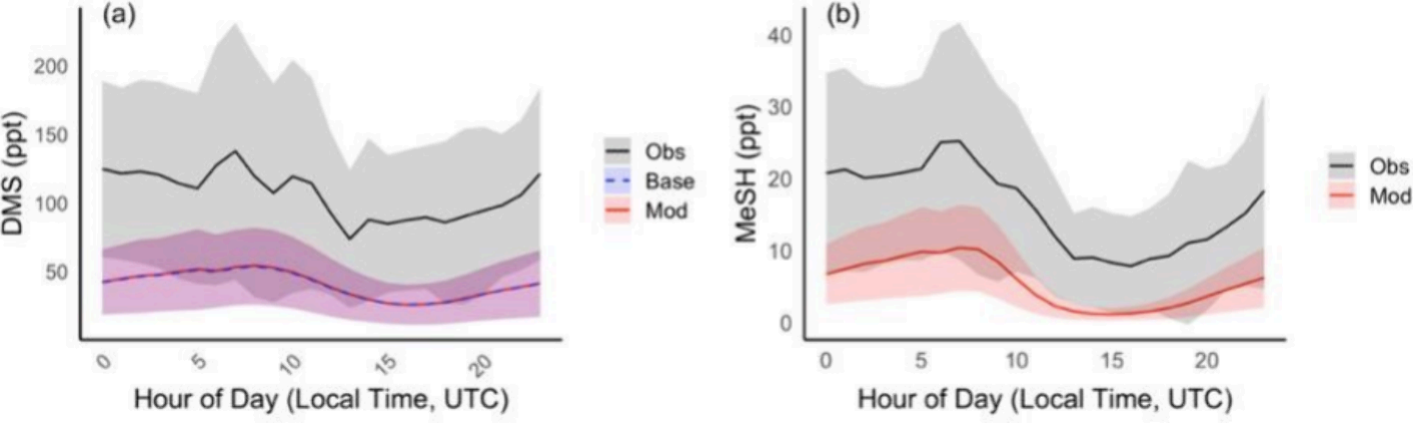
Observed and modeled diel profile of (a) DMS and (b) MeSH mixing ratios. Shading represents the standard deviation of the binned hourly mean. Location and period of measurement are from the AGENA campaign and the same as used in Figure 3.

#### 3.2.2 Comparison with Aircraft Observation

As we find an increase in global annual mean surface layer SO_2_ mixing ratios, we present a comparison of model output with ATom-4 aircraft observations^51,52^ for specific days of measurement for SO_2_ as shown in Figure 6.^46^ We use ATom-4 data as this campaign provides unique high-precision measurements of SO_2_ and other species while sampling in widespread remote marine regions of the troposphere. For this comparison, the model is sampled at the time and location of aircraft measurements by ATom-4 using the planeflight diagnostic of GEOS-Chem. SO_2_ concentrations measured during ATom-4 by laser-induced fluorescence (LIF) are shown in Figure 6 alongside MOD values for nearest neighbor grid cells across altitude levels up to 8 km. We selected a subset of the ATom-4 data set, retaining only the data points within the Pacific Ocean region where the terrestrial influence was relatively low compared to other flight regions and where our model indicates an increase in SO_2_ due to the inclusion of MeSH emissions and chemistry.^47,48^ Exact regional boundaries chosen are shown in Figure S3 (left). Over the Pacific Ocean, both BASE and MOD cases generally underestimate SO_2_ concentrations, especially at higher altitudes. This general underestimation is less severe in the MOD case due to the additional SO_2_ formation from MeSH emissions and oxidation, helping to reduce the model bias.

**Figure 6:**
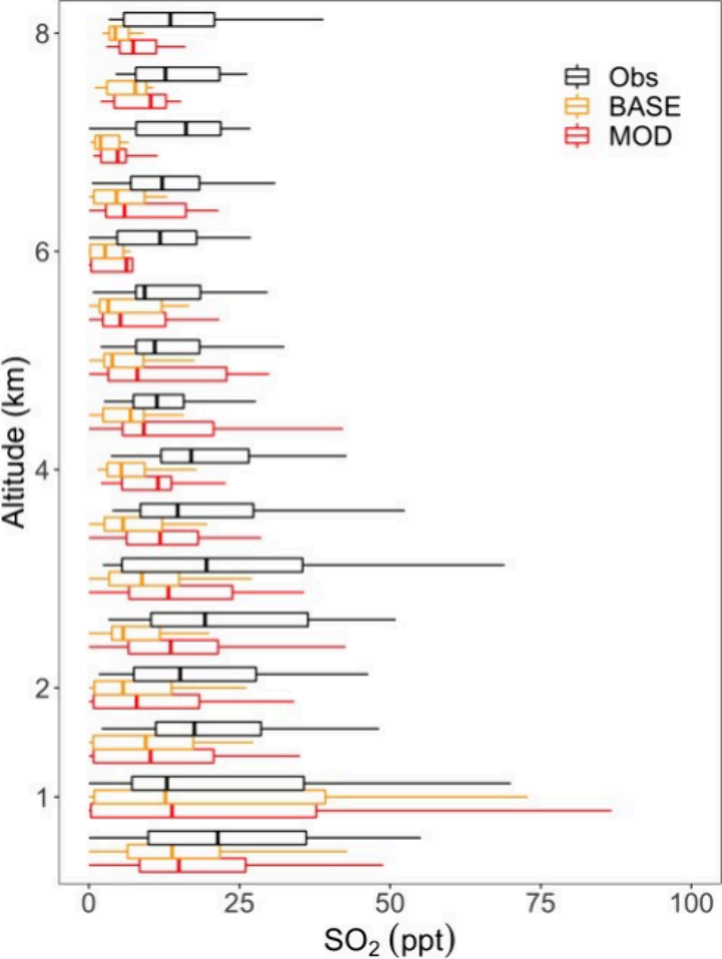
Vertical profiles of SO_2_ mixing ratios from ATom-4 observations (black) and model with simulation MOD sampled along the ATom-4 flight tracks (red) binned every 500 m of flight altitude for the Pacific Ocean region. Also shown are modeled results with DMS chemistry implemented in our previous work^17^ named as BASE in this study (orange). Box plot whiskers show a full range of distributions at each altitude bin. SO_2_ observations from ATom-4 campaign were measured by laser-induced fluorescence (LIF).

Note that SO_2_ is not the only major product of MeSH oxidation chemistry; other oxidation products of MeSH follow alternative pathways and contribute to the formation of MSA and H_2_SO_4_ as well. Overall, we can confirm that the addition of MeSH emissions and chemistry acts as an additional source of SO_2_, increasing modeled SO_2_ compared with simulation BASE for the Pacific Ocean and reducing the underprediction observed with the previously expanded DMS chemistry alone. Remaining model biases could be at least partially attributed to uncertainty in MeSH oxidation processes, along with known uncertainties in the DMS and MeSH emission inputs. Aside from these factors, additional marine sulfur species such as benzothiazole and dimethyl disulfide (DMDS) have been reported as possible sources of SO_2_ in the marine atmosphere and could help further improve model performance, a possibility deserving further investigation.^22,49,50^

### 3.3 Global Modeling of MeSH and Sulfur Budget

Simulations done in this study demonstrate the importance of MeSH emissions and oxidation processes as sources of SO_2_ and sulfate in the atmosphere. This study offers an initial modeling effort to assess how hypothetical marine MeSH emissions might influence the global sulfur cycle, recognizing the current uncertainties in MeSH emission and chemistry. However, we do highlight the global spatiotemporal variability of inferred MeSH emission flux magnitudes, as well as important uncertainties related to the *F*_DMS_:*F*_MeSH_ ratio, both of which suffer from a lack of sufficient observations. We emphasize the importance of future studies aimed at enhancing our understanding of the scale and spatial variability of MeSH emissions from the ocean.

The oxidation scheme we implement here includes only gas-phase reactions involving formation of reduced sulfur intermediates, which contribute to changes in concentrations of major oxidation products such as SO_2_ and MSA, potentially contributing to increases in sulfate concentration. With this new chemical scheme, the global annual mean surface SO_2_ concentration over the ocean increases by 12.1% relative to the BASE scheme in GEOS-Chem due to an increase in precursor concentrations eventually ending up as SO_2_. Therefore, MeSH acts as an additional source of SO_2_ from the ocean surface, contributing to a reduced bias relative to aircraft observations and helping to further develop the modeled marine sulfur budget. We also find an increase in sulfate aerosol concentrations by about 19.1% due to the additional SO_2_ and MSA, both of which can further oxidize to sulfate.

In this proposed scheme, OH, BrO, NO_3_, and Cl species act as oxidants of MeSH contributing to 86.4%, 8.4%, 4.8%, and 0.4% global annual mean surface MeSH loss, highlighting the relative importance of these loss processes in determining the surface MeSH budget (see the SI and Figure S2). We also find that at higher latitudes, gas phase oxidation of MeSH by BrO and NO_3_ (NH for NO_3_) occurs at higher rates compared to other regions, increasing the relative importance for these two sinks on regional scales in determining the budget of MeSH. Our scheme includes additional pathways for the formation of direct gas-phase MSA, which is considered an important contributor to aerosol formation, especially in the Southern Hemisphere, and which is currently underpredicted in the default version of GEOS-Chem.

The increase in surface sulfate concentration with the addition of emission and oxidation of MeSH chemistry contributes to an increase in the global annual mean surface layer sulfate aerosol mass concentration of 19.1%, largely due to the increase in gas-phase precursors SO_2_, MSA, and H_2_SO_4_.

This overall change would be expected to increase the number concentration for the nucleation mode of the aerosol.

Overall, this work highlights the potential for natural marine sulfur aerosol formation resulting from MeSH emissions and oxidation, establishing a more complete picture of the marine sulfur budget for air quality and climate models. While much work is still needed to further constrain and evaluate the uncertainties related to MeSH emissions and chemistry, this work offers a starting point for estimating and understanding the impacts of its addition as a relevant sulfur species. Additionally, it offers insights into future impacts on climate control and local air quality based on contributions to total aerosol burdens.

## Supplementary Material



## Data Availability

The observational data obtained during ATom-4 are published through the Distributed Active Archive Center for Biogeochemical Dynamics (DAAC) at 10.3334/ORNLDAAC/1890. The ATom-4 data set is also available at 10.3334/ORNLDAAC/1581. The AGENA data set is available at https://adc.arm.gov/discovery/#/results/site_code::ena/iopShortName::ena2022AGENA. Modeled output for DMS, MeSH, MSA, SO_2_, and SO_4_^2–^ can be accessed from 10.5281/zenodo.16730017.

## Notes

Sources, emission fluxes, and atmospheric fate of DMS and MeSH, strategy to include MeSH emission in the global model, literature review on modeling MeSH, model details, sensitivity tests, list of MeSH oxidation reactions, uncertainties involved in the emission fluxes of DMS and MeSH, chemical sink of MeSH (PDF)
