# Supplementary material for: Global Impacts of Marine Methanethiol Emissions and Chemistry in the Atmosphere
Source: Environ Sci Technol. 2025 Sep 18;59(38):20421–8. doi: 10.1021/acs.est.5c02019 (PMC12490017; doi:10.1021/acs.est.5c02019)
Supplement: Supplementary file 1 [file es5c02019_si_001.pdf]

Supporting Information

Global Impacts of Marine Methanethiol Emissions and Chemistry in the Atmosphere

Linia Tashmim[1], William C. Porter[1*], Timothy H. Bertram[2], Delaney B. Kilgour[2] and Andrew Rollins[3]*

[1]Department of Environmental Sciences, University of California, Riverside, CA 92521, USA
[2]Department of Chemistry, University of Wisconsin-Madison, Madison, WI 53706, USA
[3]Chemical Sciences Division, NOAA Earth System Research Laboratory, Boulder, CO 80305, USA

Correspondence: William C. Porter (william.porter@ucr.edu)

This file includes:

Supplementary Text 14 pages
Tables S1
Figures S1-S4
References 1-54

Source and emission flux of MeSH

In ocean surface waters, DMS originates from the cleavage of dimethylsulfoniopropionate (DMSP), produced by phytoplankton and other marine organisms.[1,2] Once generated, DMS in seawater can be converted to non-volatile sulfur or transformed by bacterial or photochemical process which altogether results in a seawater lifetime on the order of a few days.[3–6] Besides DMS, another volatile sulfur species, MeSH, is likewise generated in seawater through the same precursor dimethylsulfoniopropionate (DMSP) demethylation or demethiolation.[4,5,7,8] Previous work has shown that oceanic MeSH is the primary product of dissolved DMSP consumption during biological demethylation, with a substantial yield of around 75%, compared to the approximately 10% yield of DMS.[5,9,10] Although MeSH generally exhibits higher yields than DMS in the dissolved phase, rapid consumption by bacteria and phytoplankton results in significantly shorter marine lifetime for MeSH (hours) compared to DMS (days) and lower steady-state dissolved concentrations.[4] Since the recognition of the climate-relevance of DMS there has been a heightened focus on measuring surface ocean DMS concentration and tracking its eventual fate. This attention has facilitated the creation of global climatologies of dissolved DMS concentrations and emission fluxes which has been a crucial step for integrating existing observations into air quality and global chemical transport models.[2,11] In contrast, measurements of oceanic MeSH are still limited, hindering the development of robust and validated global emission flux profiles for MeSH.[6,8,12–14] A recent work introduces a global emission field of MeSH based on in situ seawater dissolved observations covering a large global area.[15]

As with seawater concentrations, measurements of atmospheric abundance of MeSH are understudied relative to DMS. Literature sources show that in the remote southwestern Pacific, MeSH mixing ratios range from <10 ppt to 65 ppt, while other observations in coastal waters west of the Antarctic Peninsula show MeSH mixing ratios up to 3.6 ppt.[16] Seawater concentration measurements in the Atlantic and Arctic Oceans, as well as air-sea flux measurement in the Southwest Pacific Ocean have also been performed, highlighting the relationships between chemical and biological dynamics and eventual MeSH concentrations.[13,14]

Strategy of developing global emission flux for MeSH

Among the limited measurements for seawater concentration and flux of MeSH, some recent examples provide critical information regarding the correlation in emission and fluxes between DMS and MeSH. One such measurement of gas-phase volatile organosulfur molecules during a mesocosm phytoplankton bloom experiment using coastal seawater reports that the DMS:MeSH molar ratio during the pre-bloom phase is low,[12] suggesting that MeSH can have a significant impact on atmospheric oxidative capacity and secondary sulfate formation in coastal environments. Another study presents direct flux measurements of MeSH using the eddy covariance (EC) method with a high-resolution proton-transfer-reaction time-of-flight mass spectrometer (PTR-ToFMS) instrument at a coastal ocean site, reporting mean mixing ratios of DMS and MeSH as 72 ppt and 19.1 ppt respectively.[8] In this study, representative of relatively clean coastal conditions due to its proximity to deep submarine canyons, the campaign mean DMS to MeSH flux ratio was reported as $F_{DMS}:F_{MeSH} = 5.5 \pm 3.0$, calculated from the ratio of 304 individual coincident measurements of F_{DMS} and F_{MeSH}. While a recent work[15] provide an important compilation of global seawater MeSH measurements where MeSH follows a linear correlation with concurrently measured DMS, the measured flux ratio[12] provides a starting point

to consider climatology base MeSH concentrations and fluxes to the atmosphere in global chemical transport model by mimicking the climatological concentration-based emission profile of DMS and applying this flux ratio to that emission rate. This provides a first-order representation, with future work aiming to incorporate latest approaches[15] into more refined emission schemes.

Fate of DMS in the atmosphere

Once emitted from the ocean, DMS oxidation in the marine atmosphere leads to formation of methanesulfonic acid (MSA), hydroperoxymethyl thioformate (HPMTF) and sulfur dioxide (SO_2).[17–21] These oxidation products can subsequently produce sulfuric acid (H_2SO_4) and secondary sulfate aerosols, which are categorized as non-sea-salt sulfate aerosols ($nssSO_4^{2-}$) in the marine environment. Each of these species play a crucial role in aerosol nucleation, CCN generation, and particle growth, impacting the marine atmosphere in many ways.[20,22–25]

Fate of MeSH in the atmosphere

Once MeSH enters the atmosphere it undergoes oxidation by primary atmospheric oxidants OH, BrO, NO_3, Cl as shown in Figure S1.[26–29] While bimolecular rate constants of these reactions have been established, there is limited existing research on reactive intermediates or yields of stable products from MeSH oxidation. However, MeSH oxidation by OH has been reported to produce the methyl thiyl radical (CH_3S) with a yield of 1.1 ± 0.2, connecting it to known reactions in the DMS hydrogen abstraction pathway.[29,30] Another recent study has reported this yield as 0.98 under low NO_x conditions at 298 K, with MeSH + OH being ten times faster compared to DMS + OH resulting efficient SO_2 production from MeSH.[31,32] Once formed, CH_3S efficiently produces sulfur dioxide (SO_2) through a series of reactions, including the reversible addition of O_2 to CH_3S, forming a methyl thiyl peroxy radical (CH_3SOO), which further oxidizes to produce SO_2.[26] Bimolecular reactions of CH_3S with O_3 and NO_2 also contribute to SO_2 production, forming CH_3SO that oxidizes further to produce SO_2.[19,30,33,34] A non-SO_2-producing reaction pathway from CH_3SO_2 + O_3 is found to form CH_3SO_3, which further reacts to produce gas phase H_2SO_4 and MSA.[30] Recent theoretical work also explores the formation possibility of CH_3SO_2OO from CH_3SO_2 through O_2 addition, as well as its subsequent contribution towards gas-phase MSA formation.[35] Once formed, CH_3SO_2OO can either form CH_3SO_3 via bimolecular reaction or participate in direct conversion to MSA or peroxides, with the latter cases believed to be relatively unlikely.[35,36] The formation and fate of CH_3SO_3 is an important part of this oxidation mechanism as it has potential to form SO_3 (eventually H_2SO_4) and MSA. This theoretical work also discusses the reaction potentials of CH_3SO_3 + HO_2, H_2O or CH_3SH and highlighted the reason why the H-abstraction via CH_3SO_3 + MeSH should be faster than that of CH_3SO_3 + HO_2. Even though there is no experimental work done yet to investigate the H-abstraction via CH_3SO_3 + MeSH, from our current understanding this reaction possess more importance for MeSH derived MSA formation than that of DMS.

Previous Modeling Approach on MeSH

Based on the link between MeSH oxidation by OH and known DMS oxidation chemistry, a MeSH oxidation mechanism has been implemented previously in a 0-D chemical box model.[8] However, this study did not include aqueous-phase loss processes for DMS and relevant intermediates, nor did it evaluate the impact of this mechanism on a global scale. The SPectral Aerosol Cloud Chemistry Interaction Model (SPACCIM) has also been used to investigate the relative importance of MeSH and DMS towards gas-phase H_2SO_4 formation.[36] Most recently, CAM-Chem model was

used to implement monthly field of global MeSH emission which was constructed by compiling a database of seawater MeSH concentration.[15] This modeling study also assesses the impact of gas-phase MeSH oxidation chemistry alongside global MeSH emissions, revealing a 30–70% increase in sulfate aerosol burden over the Southern Ocean, which enhances the aerosol cooling effect.

Model simulations and reaction choice

We here refer to simulations performed using our previously implemented custom chemical mechanism for DMS oxidation reactions,[21] which was constructed based on an integration of previous mechanisms along with further improvement following recent experimental literature updates to chemical kinetics merged with the reactions from Table S1 except the first four reactions that involves MeSH as the "BASE" mechanism.[20,37–40] This mechanism features both gas- and aqueous-phase reactions, including the formation of important intermediates such as dimethyl sulfoxide (DMSO), methanesulfinic acid (MSIA), MSA, HPMTF, and SO_2 but no heterogeneous loss of HPMTF to clouds and aerosols. We further implement and evaluate a custom chemical mechanism for MeSH oxidation (Table S1) which is integrated with our previously explored DMS oxidation mechanism and includes MeSH emission, referred to hereafter as "MOD" (Table S1). This constructed mechanism includes additional updates for reactions considering alternative fates of known intermediates according to Master Chemical Mechanism (MCM) v3.3.1.[8]

Output from MOD simulations was then compared against BASE simulations to understand the contribution of MeSH emission and oxidation reaction on the spatial pattern of the surface concentration of major oxidation products of MeSH.

As shown in Table S1, the modified MeSH chemistry simulations examined here includes only gas-phase oxidation of MeSH and its intermediate oxidation products by OH, NO_3, BrO, and Cl based on earlier proposed MeSH oxidation pathway.[8] Note that the default GEOS-Chem chemical mechanism contains detailed HO_x–NO_x–VOC–O_3–halogen tropospheric chemistry along with recently updated halogen chemistry and in-cloud processing.[17,41–45] Further building upon this previous mechanism, the Table S1also includes additional reactions based on MCMv3.3.1 and is followed by recent study which studies temperature dependent gas-phase MSA formation with key intermediates such as methylsulfonyl radical, CH_3SO_2 and its corresponding peroxy compound CH_3SO_2OO, which eventually leads to additional gas-phase MSA formation in this mechanism.[28,35,36] For the aqueous-phase reactions as part of our previously implemented custom DMS oxidation mechanism for simulation BASE, we exclude the OH loss of MSA for all of the simulations as we find it too aggressive in the model without additional tuning.[21,38] Alongside the gas-phase reactions relevant to the added MeSH oxidation mechanism contributing to the formation of SO_2 by enhancing the precursor concentration, the default version of GEOS-Chem used here also includes in-cloud oxidation of SO_2 by H_2O_2, O_3, and O_2 catalyzed by transition metals (Mn, Fe), as well as the loss of dissolved SO_2 by HOBr and HOCl, all of which contribute to sulfate production.[17,46]

We run one simulation each for the BASE and MOD cases covering the year 2019, which was chosen to match the observation period for the eddy covariance flux measurements upon which the F_{DMS}:F_{MeSH} flux ratio used for this study was based. Model output was used to examine the burden and lifetime of MeSH and its major oxidation products along with relative contributions of each reaction pathways towards global chemical losses. Note that here the BASE simulation includes updated DMS oxidation chemistry implemented in our previous work along with the

CH_3SO_3 chemistry, while the MOD simulation also includes the additional MeSH emissions and oxidation mechanism described in this work.[21] Beyond these cases, to compare the SO_2 modeled output with aircraft observation from the NASA Atmospheric Tomography flight campaign (ATom-4), simulations were also performed for the year of 2018 to coincide with the flight campaign period. Finally, to further evaluate modeled output through a comparison with measurement of DMS and MeSH from the Aerosol Growth in the Eastern North Atlantic (AGENA) campaign, additional simulations were performed for the period of June 1, 2022 to July 15, 2022. All of these simulations were performed using a $4^{\circ} \times 5^{\circ}$ horizontal resolution, 47 vertical levels, and the GEOS-FP data product as meteorological input. In our simulations including MeSH emissions and chemistry, the added compound undergoes chemical loss and transport assuming physical properties as an advected chemical species similar to DMS, but does not undergo dry or wet deposition.

Modeled DMS and MeSH emission fluxes and uncertainties

Given our emissions assumptions, we find that both DMS and MeSH follow a similar geographic distribution and peak areas of emission in general (Figure 2). With the modeled DMS concentration climatology and parameterizations for the sea/air gas transfer velocity and climatological wind fields, we find that DMS transfers from ocean to atmosphere at the rate of 1.92×10^{-12} kg m^{-2} s^{-1}. For MeSH this emission rate drops to 3.98×10^{-13} kg m^{-2} s^{-1} to maintain the target ratio of $F_{DMS}:F_{MeSH} = 5$ implemented in this work.

Here we must acknowledge major uncertainties regarding DMS emissions used in the model as described in our previous work[21], all of which also apply to our derived MeSH emissions. We further note the need for improved inventories for both species, which will certainly play a role in subsequent oxidation product comparisons. Adding MeSH emissions derived from existing DMS emission inventories can be considered only a preliminary step towards the full addition of MeSH in global chemical transport models whereas recent work propose more realistic way of dealing with MeSH emission on a global scale.[15] Meteorological and biological drivers will certainly strongly influence the emission pattern, with coastal dynamics also potentially disproportionately contributing to spatial variability of these emissions. It should also be noted that the emission flux ratio we use here to scale MeSH emissions based on DMS inventories is based on one study from a coastal ocean site which has been frequently utilized for studying trace gas exchange between the ocean and atmosphere,[8,47–49] a limitation that introduces possible bias into our hypothetical globally extrapolated emissions. To date, there are very few measurements of DMS to MeSH flux ratio to verify how representative this applied ratio is in a global context. Measurements have shown that molar and flux ratios do vary by location, and also based on the stage of bloom and decay.[6,8,12] Therefore, to improve emission pattern accuracy in the model and support the development of the oxidation chemical mechanism, we emphasize the need for more comprehensive global MeSH emissions measurements to refine and build upon these results. Despite this limitation, our implemented approach gives an initial estimate of expected atmospheric outcomes under a feasible emissions scenario. Various non-oceanic sources of MeSH emissions have also been reported, and based on their estimated magnitudes those sources could play an important role in shaping local or regional air quality due to MeSH oxidation chemistry,[12,50] representing another known data gap and recommendation for future research efforts. Episodically high MeSH levels have been observed in urban areas, likely from local anthropogenic sources.[51,52] Therefore emission of MeSH from distinct terrestrial sources could result in weaker correlations between DMS and MeSH.[53]

Global Chemical Loss of MeSH:

Figure S2 shows that in simulation MOD, MeSH is mainly oxidized by OH in the gas phase, with 86.4% of global average loss. NO_3 oxidation of MeSH accounts for another 8.4% of global MeSH chemical losses. Over the ocean, the NO_3 loss pathway is strongest in the Northern Hemispheric coastal regions. As with DMS, this loss via NO_3 can be linked to the outflow of NO_x sources.[21,37] Oxidation by BrO is responsible for 4.8% of the global MeSH removal, with regional contribution reaching up to 30%–40% over high latitudes of the Southern Hemisphere due to high abundance of BrO in those regions compared to OH and NO_3 increases the relative importance for this sink of MeSH. Note that due to the short atmospheric lifetime of MeSH (relative to DMS), all MeSH oxidation occurs within the surface model layer, while the relatively longer lifetime of DMS allows a fraction of it to be oxidized higher up, in the lower free troposphere.

The Cl oxidation reaction contributes only about 0.4% of the overall chemical removal of MeSH, and is the least important among all modeled sinks. We also perform a sensitivity test by adding the heterogeneous loss of HPMTF to clouds, as explained in our previous work, and find that these loss processes have no impact in changing the relative contribution of sinks for MeSH.

Table S1. Overview of the MeSH oxidation mechanism. Note that, here we only listed the reactions that were added explicitly for the MeSH oxidation mechanism in the model for simulation MOD whereas Figure S1 shows some additional intermediate oxidation pathways that is common between DMS and MeSH and is part of our previously implemented DMS oxidation mechanism used in the BASE simulation and therefore not listed here.

**Gas-phase reactions**	**Rate constant (cm^3 $molecule^{-1}$ s^{-1})**	**References**
$CH_3SH + OH \rightarrow CH_3S + H_2O$	$9.90\times10^{-12}exp(356/T)$	IUPAC SOx21 (upd. 2006)
$CH_3SH + BrO \rightarrow CH_3S + HOBr$	$2.20\times10^{-15}exp(827/T)$	Ref[27]
$CH_3SH + NO_3 \rightarrow CH_3S + HNO_3$	9.20×10^{-13}	IUPAC SOx32 (upd. 2006)
$CH_3SH + Cl \rightarrow CH_3S + HCl$	$1.20\times10^{-10}exp(150/T)$	IUPAC SOx12 (upd. 2006)
$CH_3SO + O_2 \rightarrow CH_3SO_3$	$3.12\times10^{-16}exp(1580/T)$	MCMv3.3.1, Ref[8]
$CH_3SO_3 \rightarrow CH_3O_2 + H_2SO_4$*	$3.00\times10^{+13}$ $exp(-9897/T)$	Ref[35]
$CH_3SOO + NO \rightarrow NO_2 + CH_3SO$	1.10×10^{-11}	MCMv3.3.1, Ref[8,54]
$CH_3SOO + NO_2 \rightarrow NO_3 + CH_3SO$	2.20×10^{-11}	MCMv3.3.1, Ref[8]
$CH_3SOO \rightarrow CH_3SO_2$*	$7.00\times10^{+14}$ $exp(-9659/T)$	Ref[35]
$CH_3SO_2 \rightarrow CH_3O_2 + SO_2$*	$1.70\times10^{+15}exp(-8.4\times10^{+3}/T)$ $exp(1.8\times10^{+6}/T^3)$	Ref[35]
$CH_3SO_2 + O_2 \rightarrow CH_3SO_2OO$	$1.20\times10^{-16}exp(1580/T)$	Ref[35]
$CH_3SO_2OO \rightarrow CH_3SO_2$	$(1.8\times10^{-13})/(1.03\times10^{-7}\times$ $exp(7.39\times10^{+3}/T))$	Ref[35]
$CH_3SO_2OO + NO_2 \rightarrow CH_3SO_4NO_2$	$1.20\times10^{-12}(T/300)^{-0.9}$	MCMv3.3.1
$CH_3SO_2OO + NO_3 \rightarrow CH_3SO_3 + NO_2$	4.00×10^{-12}	MCMv3.3.1
$CH_3SO_2OO + NO \rightarrow CH_3SO_3 + NO_2$	1.00×10^{-11}	MCMv3.3.1
$CH_3SO_2OO + HO_2 \rightarrow 0.41CH_3SO_2OOH + 0.44CH_3SO_3 + 0.44OH + 0.15MSA + 0.15O_3$	1.00×10^{-11}	MCMv3.3.1
$CH_3SO_2OO + CH_3O_2 \rightarrow 0.3MSA + 0.7CH_3SO_3$	1.00×10^{-11}	MCMv3.3.1
$CH_3SO_4NO_2 + OH \rightarrow CH_3SO_2OO + HNO_3$	3.60×10^{-13}	MCMv3.3.1, Ref[35]
$CH_3SO_4NO_2 \rightarrow CH_3SO_2OO + NO_2$	$5.40\times10^{+16}exp(-13112/T)$	MCMv3.3.1, Ref[35]
$CH_3SO_2OOH + OH \rightarrow CH_3SO_2OO + H_2O$	3.60×10^{-12}	MCMv3.3.1, Ref[35]

*The reactions marked with asterisk (*) follows a different rate in[21] and the references therein.

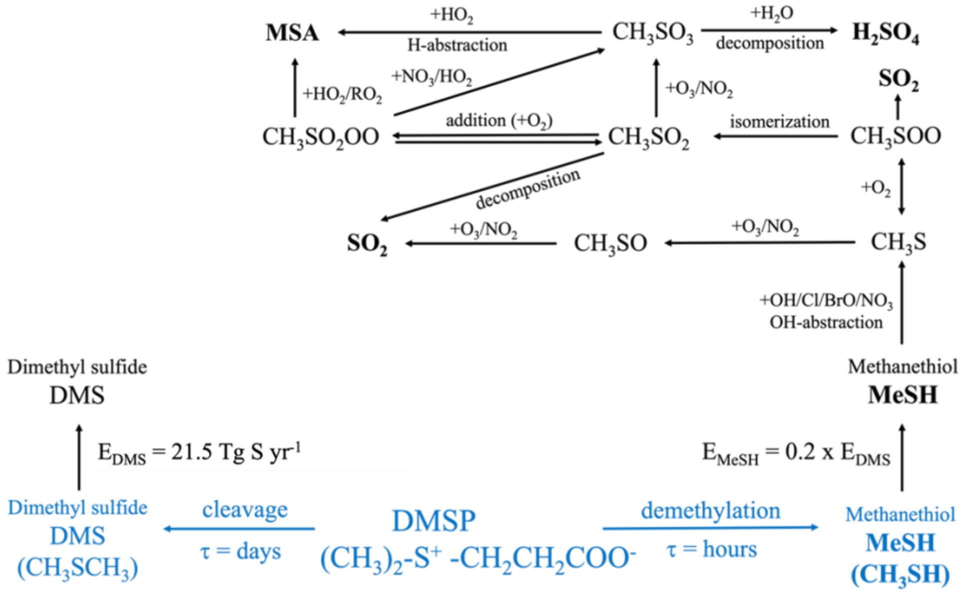

Figure S1. MeSH oxidation mechanism used in this work (simulation MOD) showing the pathway towards the formation of SO_2, H_2SO_4 and MSA in gas-phase. The major oxidation products are shown in bold. The blue arrows and text represent reactions in the seawater surface layer. The symbol τ represents the lifetime for DMS and MeSH. E_{DMS} and E_{MeSH} indicates emission of DMS and MeSH respectively in Tg S yr^{-1} unit.

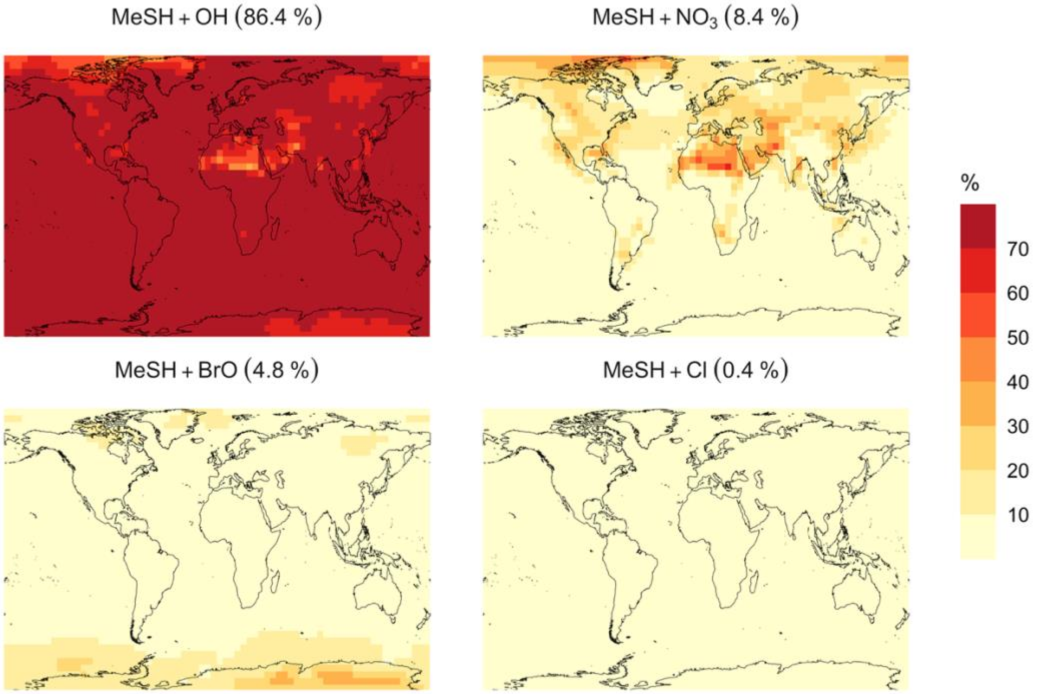

Figure S2. Geographic distribution of the annual mean surface layer fraction of total MeSH oxidation (percent) attributed to different tropospheric oxidants for simulation MOD. Percentages in parentheses indicate the average contribution to global chemical loss for the fraction of MeSH emitted for each reaction pathways presented here.

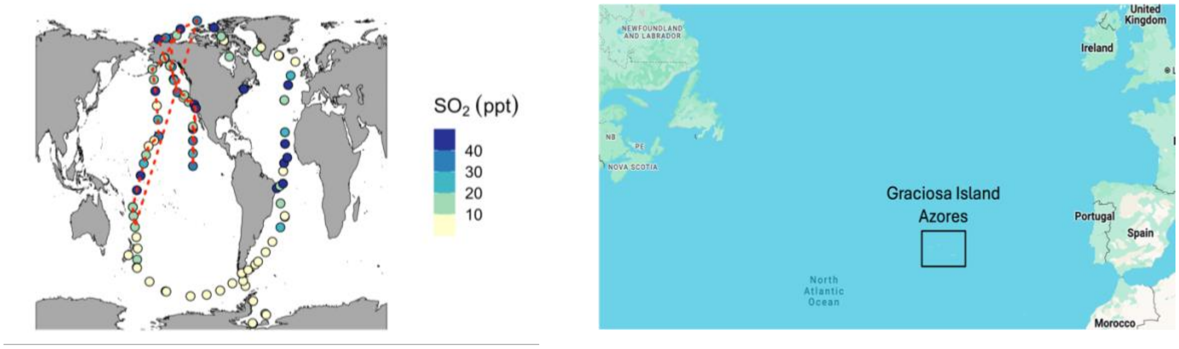

Figure S3. On the left, measurements of SO_2 during the ATom-4 mission are displayed, with the red dashed polygon outlining the region defined as the Pacific Ocean, which was used for comparison with modeled output in Figure 3. To the right, location for measurement of DMS and MeSH are shown from the Aerosol Growth in the Eastern North Atlantic (AGENA) campaign which sampled at Eastern North Atlantic site on Graciosa Island, in the Azores (39.0916°N, 28.0257°W).

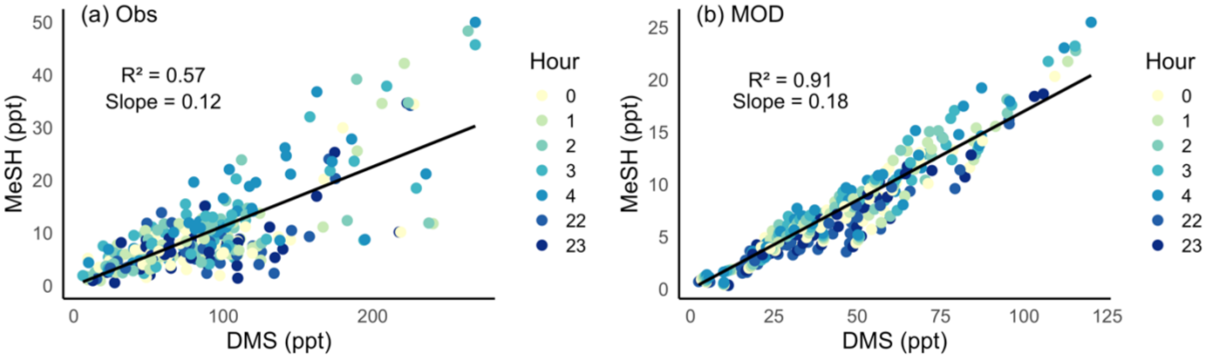

Figure S4. Correlation of DMS and MeSH mixing ratios at hourly averaged time colored by hour of day of the measurement for selective hours. Hour of day is in local time (UTC – 0). The linear least square best fit is plotted as solid black line.

REFERENCES

(1) Challenger, F.; Simpson, M. I. 320. Studies on Biological Methylation. Part XII. A Precursor of the Dimethyl Sulphide Evolved by Polysiphonia Fastigiata. Dimethyl-2-Carboxyethylsulphonium Hydroxide and Its Salts. *J. Chem. Soc.* **1948**, No. 0, 1591–1597. https://doi.org/10.1039/JR9480001591.

(2) Lana, A.; Bell, T. G.; Simó, R.; Vallina, S. M.; Ballabrera-Poy, J.; Kettle, A. J.; Dachs, J.; Bopp, L.; Saltzman, E. S.; Stefels, J.; Johnson, J. E.; Liss, P. S. An Updated Climatology of Surface Dimethlysulfide Concentrations and Emission Fluxes in the Global Ocean. *Global Biogeochemical Cycles* **2011**, *25* (1). https://doi.org/10.1029/2010GB003850.

(3) Flöck, O. R.; Andreae, M. O. Photochemical and Non-Photochemical Formation and Destruction of Carbonyl Sulfide and Methyl Mercaptan in Ocean Waters. *Marine Chemistry* **1996**, *54* (1), 11–26. https://doi.org/10.1016/0304-4203(96)00027-8.

(4) Kiene, R. P. Production of Methanethiol from Dimethylsulfoniopropionate in Marine Surface Waters. *Marine Chemistry* **1996**, *54* (1), 69–83. https://doi.org/10.1016/0304-4203(96)00006-0.

(5) Kiene, R. P.; Linn, L. J. The Fate of Dissolved Dimethylsulfoniopropionate (DMSP) in Seawater: Tracer Studies Using 35S-DMSP. *Geochimica et Cosmochimica Acta* **2000**, *64* (16), 2797–2810. https://doi.org/10.1016/S0016-7037(00)00399-9.

(6) Lawson, S. J.; Law, C. S.; Harvey, M. J.; Bell, T. G.; Walker, C. F.; de Bruyn, W. J.; Saltzman, E. S. Methanethiol, Dimethyl Sulfide and Acetone over Biologically Productive Waters in the Southwest Pacific Ocean. *Atmospheric Chemistry and Physics* **2020**, *20* (5), 3061–3078. https://doi.org/10.5194/acp-20-3061-2020.

(7) Yoch, D. C. Dimethylsulfoniopropionate: Its Sources, Role in the Marine Food Web, and Biological Degradation to Dimethylsulfide. *Applied and Environmental Microbiology* **2002**, *68* (12), 5804–5815. https://doi.org/10.1128/AEM.68.12.5804-5815.2002.

(8) Novak, G. A.; Kilgour, D. B.; Jernigan, C. M.; Vermeuel, M. P.; Bertram, T. H. Oceanic Emissions of Dimethyl Sulfide and Methanethiol and Their Contribution to Sulfur Dioxide Production in the Marine Atmosphere. *Atmospheric Chemistry and Physics* **2022**, *22* (9), 6309–6325. https://doi.org/10.5194/acp-22-6309-2022.

(9) Kettle, A. J.; Rhee, T. S.; von Hobe, M.; Poulton, A.; Aiken, J.; Andreae, M. O. Assessing the Flux of Different Volatile Sulfur Gases from the Ocean to the Atmosphere. *Journal of Geophysical Research: Atmospheres* **2001**, *106* (D11), 12193–12209. https://doi.org/10.1029/2000JD900630.

(10) Kiene, R. P.; Linn, L. J. Distribution and Turnover of Dissolved DMSP and Its Relationship with Bacterial Production and Dimethylsulfide in the Gulf of Mexico. *Limnology and Oceanography* **2000**, *45* (4), 849–861. https://doi.org/10.4319/lo.2000.45.4.0849.

(11) Galí, M.; Levasseur, M.; Devred, E.; Simó, R.; Babin, M. Sea-Surface Dimethylsulfide (DMS) Concentration from Satellite Data at Global and Regional Scales. *Biogeosciences* **2018**, *15* (11), 3497–3519. https://doi.org/10.5194/bg-15-3497-2018.

(12) Kilgour, D. B.; Novak, G. A.; Sauer, J. S.; Moore, A. N.; Dinasquet, J.; Amiri, S.; Franklin, E. B.; Mayer, K.; Winter, M.; Morris, C. K.; Price, T.; Malfatti, F.; Crocker, D. R.; Lee, C.; Cappa, C. D.; Goldstein, A. H.; Prather, K. A.; Bertram, T. H. Marine Gas-Phase Sulfur Emissions during an Induced Phytoplankton Bloom. *Atmospheric Chemistry and Physics* **2022**, *22* (2), 1601–1613. https://doi.org/10.5194/acp-22-1601-2022.

(13) Rocco, M.; Dunne, E.; Saint-Macary, A.; Peltola, M.; Barthelmeß, T.; Barr, N.; Safi, K.; Marriner, A.; Deppeler, S.; Harnwell, J.; Engel, A.; Colomb, A.; Saiz-Lopez, A.; Harvey, M.; Law, C. S.; Sellegri, K. Air-Sea Fluxes of Dimethyl Sulphide and Methanethiol in the South-West Pacific. *EGUsphere* **2023**, 1–31. https://doi.org/10.5194/egusphere-2023-516.

(14) Gros, V.; Bonsang, B.; Sarda-Estève, R.; Nikolopoulos, A.; Metfies, K.; Wietz, M.; Peeken, I. Concentrations of Dissolved Dimethyl Sulfide (DMS), Methanethiol and Other Trace Gases in Context of Microbial Communities from the Temperate Atlantic to the Arctic Ocean. *Biogeosciences* **2023**, *20* (4), 851–867. https://doi.org/10.5194/bg-20-851-2023.

(15) Wohl, C.; Villamayor, J.; Galí, M.; Mahajan, A. S.; Fernández, R. P.; Cuevas, C. A.; Bossolasco, A.; Li, Q.; Kettle, A. J.; Williams, T.; Sarda-Esteve, R.; Gros, V.; Simó, R.; Saiz-Lopez, A. Marine Emissions of Methanethiol Increase Aerosol Cooling in the Southern Ocean. *Science Advances* **2024**, *10* (48), eadq2465. https://doi.org/10.1126/sciadv.adq2465.

(16) Berresheim, H. Biogenic Sulfur Emissions from the Subantarctic and Antarctic Oceans. *Journal of Geophysical Research: Atmospheres* **1987**, *92* (D11), 13245–13262. https://doi.org/10.1029/JD092iD11p13245.

(17) Chen, Q.; Schmidt, J. A.; Shah, V.; Jaeglé, L.; Sherwen, T.; Alexander, B. Sulfate Production by Reactive Bromine: Implications for the Global Sulfur and Reactive Bromine Budgets. *Geophysical Research Letters* **2017**, *44* (13), 7069–7078. https://doi.org/10.1002/2017GL073812.

(18) Faloona, I. Sulfur Processing in the Marine Atmospheric Boundary Layer: A Review and Critical Assessment of Modeling Uncertainties. *Atmospheric Environment* **2009**, *43* (18), 2841–2854. https://doi.org/10.1016/j.atmosenv.2009.02.043.

(19) Hoffmann, E. H.; Tilgner, A.; Schrödner, R.; Bräuer, P.; Wolke, R.; Herrmann, H. An Advanced Modeling Study on the Impacts and Atmospheric Implications of Multiphase Dimethyl Sulfide Chemistry. *Proceedings of the National Academy of Sciences* **2016**, *113* (42), 11776–11781. https://doi.org/10.1073/pnas.1606320113.

(20) Novak, G. A.; Fite, C. H.; Holmes, C. D.; Veres, P. R.; Neuman, J. A.; Faloona, I.; Thornton, J. A.; Wolfe, G. M.; Vermeuel, M. P.; Jernigan, C. M.; Peischl, J.; Ryerson, T. B.; Thompson, C. R.; Bourgeois, I.; Warneke, C.; Gkatzelis, G. I.; Coggon, M. M.; Sekimoto, K.; Bui, T. P.; Dean-Day, J.; Diskin, G. S.; DiGangi, J. P.; Nowak, J. B.; Moore, R. H.; Wiggins, E. B.; Winstead, E. L.; Robinson, C.; Thornhill, K. L.; Sanchez, K. J.; Hall, S. R.; Ullmann, K.; Dollner, M.; Weinzierl, B.; Blake, D. R.; Bertram, T. H. Rapid Cloud Removal of Dimethyl Sulfide Oxidation Products Limits SO2 and Cloud Condensation Nuclei Production in the Marine Atmosphere. *Proceedings of the National Academy of Sciences* **2021**, *118* (42), e2110472118. https://doi.org/10.1073/pnas.2110472118.

(21) Tashmim, L.; Porter, W. C.; Chen, Q.; Alexander, B.; Fite, C. H.; Holmes, C. D.; Pierce, J. R.; Croft, B.; Ishino, S. Contribution of Expanded Marine Sulfur Chemistry to the Seasonal Variability of Dimethyl Sulfide Oxidation Products and Size-Resolved Sulfate Aerosol. *Atmospheric Chemistry and Physics* **2024**, *24* (6), 3379–3403. https://doi.org/10.5194/acp-24-3379-2024.

(22) Carslaw, K. S.; Lee, L. A.; Reddington, C. L.; Pringle, K. J.; Rap, A.; Forster, P. M.; Mann, G. W.; Spracklen, D. V.; Woodhouse, M. T.; Regayre, L. A.; Pierce, J. R. Large Contribution of Natural Aerosols to Uncertainty in Indirect Forcing. *Nature* **2013**, *503* (7474), 67–71. https://doi.org/10.1038/nature12674.

(23) Thomas, M. A.; Suntharalingam, P.; Pozzoli, L.; Rast, S.; Devasthale, A.; Kloster, S.; Feichter, J.; Lenton, T. M. Quantification of DMS Aerosol-Cloud-Climate Interactions Using the ECHAM5-HAMMOZ Model in a Current Climate Scenario. *Atmospheric Chemistry and Physics* **2010**, *10* (15), 7425–7438. https://doi.org/10.5194/acp-10-7425-2010.
(24) von Glasow, R.; Crutzen, P. J. Model Study of Multiphase DMS Oxidation with a Focus on Halogens. *Atmospheric Chemistry and Physics* **2004**, *4* (3), 589–608. https://doi.org/10.5194/acp-4-589-2004.
(25) Schobesberger, S.; Junninen, H.; Bianchi, F.; Lönn, G.; Ehn, M.; Lehtipalo, K.; Dommen, J.; Ehrhart, S.; Ortega, I. K.; Franchin, A.; Nieminen, T.; Riccobono, F.; Hutterli, M.; Duplissy, J.; Almeida, J.; Amorim, A.; Breitenlechner, M.; Downard, A. J.; Dunne, E. M.; Flagan, R. C.; Kajos, M.; Keskinen, H.; Kirkby, J.; Kupc, A.; Kürten, A.; Kurtén, T.; Laaksonen, A.; Mathot, S.; Onnela, A.; Praplan, A. P.; Rondo, L.; Santos, F. D.; Schallhart, S.; Schnitzhofer, R.; Sipilä, M.; Tomé, A.; Tsagkogeorgas, G.; Vehkamäki, H.; Wimmer, D.; Baltensperger, U.; Carslaw, K. S.; Curtius, J.; Hansel, A.; Petäjä, T.; Kulmala, M.; Donahue, N. M.; Worsnop, D. R. Molecular Understanding of Atmospheric Particle Formation from Sulfuric Acid and Large Oxidized Organic Molecules. *Proceedings of the National Academy of Sciences* **2013**, *110* (43), 17223–17228. https://doi.org/10.1073/pnas.1306973110.
(26) Butkovskaya, N. I.; Setser, D. W. Reactions of OH and OD Radicals with Simple Thiols and Sulfides Studied by Infrared Chemiluminescence of Isotopic Water Products: Reaction OH + CH3SH Revisited. *International Journal of Chemical Kinetics* **2021**, *53* (6), 702–715. https://doi.org/10.1002/kin.21475.
(27) Aranda, A.; Salgado, S. Kinetic and Products of the BrO+CH3SH Reaction: Temperature and Pressure Dependence. *Chemical Physics Letters* **2002**, *357*, 471–476. https://doi.org/10.1016/S0009-2614(02)00561-4.
(28) Burkholder, J. B.; Sander, S. P.; Abbatt, J. P. D.; Barker, J. R.; Cappa, C.; Crounse, J. D.; Dibble, T. S.; Huie, R. E.; Kolb, C. E.; Kurylo, M. J.; Orkin, V. L.; Percival, C. J.; Wilmouth, D. M.; Wine, P. H. Chemical Kinetics and Photochemical Data for Use in Atmospheric Studies; Evaluation Number 19, 2020. https://dataverse.jpl.nasa.gov/dataset.xhtml?persistentId=hdl:2014/49199 (accessed 2023-05-17).
(29) Tyndall, G. S.; Ravishankara, A. R. Kinetics of the Reaction of the Methylthio Radical with Ozone at 298 K. *J. Phys. Chem.* **1989**, *93* (12), 4707–4710. https://doi.org/10.1021/j100349a006.
(30) Barnes, I.; Hjorth, J.; Mihalopoulos, N. Dimethyl Sulfide and Dimethyl Sulfoxide and Their Oxidation in the Atmosphere. *Chem. Rev.* **2006**, *106* (3), 940–975. https://doi.org/10.1021/cr020529+.
(31) Chen, J.; Berndt, T.; Møller, K. H.; Lane, J. R.; Kjaergaard, H. G. Atmospheric Fate of the CH3SOO Radical from the CH3S + O2 Equilibrium. *J. Phys. Chem. A* **2021**, *125* (40), 8933–8941. https://doi.org/10.1021/acs.jpca.1c06900.
(32) Mai, T. V.-T.; Nguyen, H. T.; Huynh, L. K. Kinetics of Hydrogen Abstraction from CH3SH by OH Radicals: An *Ab Initio* RRKM-Based Master Equation Study. *Atmospheric Environment* **2020**, *242*, 117833. https://doi.org/10.1016/j.atmosenv.2020.117833.
(33) Lucas, D. D.; Prinn, R. G. Mechanistic Studies of Dimethylsulfide Oxidation Products Using an Observationally Constrained Model. *Journal of Geophysical Research:*

Atmospheres **2002**, *107* (D14), ACH 12-1-ACH 12-26. https://doi.org/10.1029/2001JD000843.
(34) Mardyukov, A.; Schreiner, P. R. Atmospherically Relevant Radicals Derived from the Oxidation of Dimethyl Sulfide. *Acc. Chem. Res.* **2018**, *51* (2), 475–483. https://doi.org/10.1021/acs.accounts.7b00536.
(35) Chen, J.; Lane, J. R.; Bates, K. H.; Kjaergaard, H. G. Atmospheric Gas-Phase Formation of Methanesulfonic Acid. *Environ. Sci. Technol.* **2023**, *57* (50), 21168–21177. https://doi.org/10.1021/acs.est.3c07120.
(36) Berndt, T.; Hoffmann, E. H.; Tilgner, A.; Stratmann, F.; Herrmann, H. Direct Sulfuric Acid Formation from the Gas-Phase Oxidation of Reduced-Sulfur Compounds. *Nat Commun* **2023**, *14* (1), 4849. https://doi.org/10.1038/s41467-023-40586-2.
(37) Chen, Q.; Sherwen, T.; Evans, M.; Alexander, B. DMS Oxidation and Sulfur Aerosol Formation in the Marine Troposphere: A Focus on Reactive Halogen and Multiphase Chemistry. *Atmospheric Chemistry and Physics* **2018**, *18* (18), 13617–13637. https://doi.org/10.5194/acp-18-13617-2018.
(38) Fung, K. M.; Heald, C. L.; Kroll, J. H.; Wang, S.; Jo, D. S.; Gettelman, A.; Lu, Z.; Liu, X.; Zaveri, R. A.; Apel, E. C.; Blake, D. R.; Jimenez, J.-L.; Campuzano-Jost, P.; Veres, P. R.; Bates, T. S.; Shilling, J. E.; Zawadowicz, M. Exploring Dimethyl Sulfide (DMS) Oxidation and Implications for Global Aerosol Radiative Forcing. *Atmospheric Chemistry and Physics* **2022**, *22* (2), 1549–1573. https://doi.org/10.5194/acp-22-1549-2022.
(39) Veres, P. R.; Neuman, J. A.; Bertram, T. H.; Assaf, E.; Wolfe, G. M.; Williamson, C. J.; Weinzierl, B.; Tilmes, S.; Thompson, C. R.; Thames, A. B.; Schroder, J. C.; Saiz-Lopez, A.; Rollins, A. W.; Roberts, J. M.; Price, D.; Peischl, J.; Nault, B. A.; Møller, K. H.; Miller, D. O.; Meinardi, S.; Li, Q.; Lamarque, J.-F.; Kupc, A.; Kjaergaard, H. G.; Kinnison, D.; Jimenez, J. L.; Jernigan, C. M.; Hornbrook, R. S.; Hills, A.; Dollner, M.; Day, D. A.; Cuevas, C. A.; Campuzano-Jost, P.; Burkholder, J.; Bui, T. P.; Brune, W. H.; Brown, S. S.; Brock, C. A.; Bourgeois, I.; Blake, D. R.; Apel, E. C.; Ryerson, T. B. Global Airborne Sampling Reveals a Previously Unobserved Dimethyl Sulfide Oxidation Mechanism in the Marine Atmosphere. *Proceedings of the National Academy of Sciences* **2020**, *117* (9), 4505–4510. https://doi.org/10.1073/pnas.1919344117.
(40) Cala, B. A.; Archer-Nicholls, S.; Weber, J.; Abraham, N. L.; Griffiths, P. T.; Jacob, L.; Shin, Y. M.; Revell, L. E.; Woodhouse, M.; Archibald, A. T. Development, Intercomparison, and Evaluation of an Improved Mechanism for the Oxidation of Dimethyl Sulfide in the UKCA Model. *Atmospheric Chemistry and Physics* **2023**, *23* (23), 14735–14760. https://doi.org/10.5194/acp-23-14735-2023.
(41) Bey, I.; Jacob, D. J.; Yantosca, R. M.; Logan, J. A.; Field, B. D.; Fiore, A. M.; Li, Q.; Liu, H. Y.; Mickley, L. J.; Schultz, M. G. Global Modeling of Tropospheric Chemistry with Assimilated Meteorology: Model Description and Evaluation. *Journal of Geophysical Research: Atmospheres* **2001**, *106* (D19), 23073–23095. https://doi.org/10.1029/2001JD000807.
(42) Holmes, C. D.; Bertram, T. H.; Confer, K. L.; Graham, K. A.; Ronan, A. C.; Wirks, C. K.; Shah, V. The Role of Clouds in the Tropospheric NOx Cycle: A New Modeling Approach for Cloud Chemistry and Its Global Implications. *Geophysical Research Letters* **2019**, *46* (9), 4980–4990. https://doi.org/10.1029/2019GL081990.
(43) Parrella, J. P.; Jacob, D. J.; Liang, Q.; Zhang, Y.; Mickley, L. J.; Miller, B.; Evans, M. J.; Yang, X.; Pyle, J. A.; Theys, N.; Van Roozendael, M. Tropospheric Bromine Chemistry:

Implications for Present and Pre-Industrial Ozone and Mercury. *Atmospheric Chemistry and Physics* **2012**, *12* (15), 6723–6740. https://doi.org/10.5194/acp-12-6723-2012.
(44) Schmidt, J. A.; Jacob, D. J.; Horowitz, H. M.; Hu, L.; Sherwen, T.; Evans, M. J.; Liang, Q.; Suleiman, R. M.; Oram, D. E.; Le Breton, M.; Percival, C. J.; Wang, S.; Dix, B.; Volkamer, R. Modeling the Observed Tropospheric BrO Background: Importance of Multiphase Chemistry and Implications for Ozone, OH, and Mercury. *Journal of Geophysical Research: Atmospheres* **2016**, *121* (19), 11,819-11,835. https://doi.org/10.1002/2015JD024229.
(45) Wang, X.; Jacob, D. J.; Eastham, S. D.; Sulprizio, M. P.; Zhu, L.; Chen, Q.; Alexander, B.; Sherwen, T.; Evans, M. J.; Lee, B. H.; Haskins, J. D.; Lopez-Hilfiker, F. D.; Thornton, J. A.; Huey, G. L.; Liao, H. The Role of Chlorine in Global Tropospheric Chemistry. *Atmospheric Chemistry and Physics* **2019**, *19* (6), 3981–4003. https://doi.org/10.5194/acp-19-3981-2019.
(46) Wang, X.; Jacob, D. J.; Downs, W.; Zhai, S.; Zhu, L.; Shah, V.; Holmes, C. D.; Sherwen, T.; Alexander, B.; Evans, M. J.; Eastham, S. D.; Neuman, J. A.; Veres, P. R.; Koenig, T. K.; Volkamer, R.; Huey, L. G.; Bannan, T. J.; Percival, C. J.; Lee, B. H.; Thornton, J. A. Global Tropospheric Halogen (Cl, Br, I) Chemistry and Its Impact on Oxidants. *Atmospheric Chemistry and Physics* **2021**, *21* (18), 13973–13996. https://doi.org/10.5194/acp-21-13973-2021.
(47) Kim, M. J.; Novak, G. A.; Zoerb, M. C.; Yang, M.; Blomquist, B. W.; Huebert, B. J.; Cappa, C. D.; Bertram, T. H. Air-Sea Exchange of Biogenic Volatile Organic Compounds and the Impact on Aerosol Particle Size Distributions. *Geophysical Research Letters* **2017**, *44* (8), 3887–3896. https://doi.org/10.1002/2017GL072975.
(48) Novak, G. A.; Vermeuel, M. P.; Bertram, T. H. Simultaneous Detection of Ozone and Nitrogen Dioxide by Oxygen Anion Chemical Ionization Mass Spectrometry: A Fast-Time-Response Sensor Suitable for Eddy Covariance Measurements. *Atmospheric Measurement Techniques* **2020**, *13* (4), 1887–1907. https://doi.org/10.5194/amt-13-1887-2020.
(49) Vermeuel, M. P.; Novak, G. A.; Jernigan, C. M.; Bertram, T. H. Diel Profile of Hydroperoxymethyl Thioformate: Evidence for Surface Deposition and Multiphase Chemistry. *Environ. Sci. Technol.* **2020**, *54* (19), 12521–12529. https://doi.org/10.1021/acs.est.0c04323.
(50) Galí, M.; Devred, E.; Babin, M.; Levasseur, M. Decadal Increase in Arctic Dimethylsulfide Emission. *Proceedings of the National Academy of Sciences* **2019**, *116* (39), 19311–19317. https://doi.org/10.1073/pnas.1904378116.
(51) Deng, K.; Huo, J.; Wang, Y.; Wang, L.; Yin, S.; Li, C.; Li, Y.; Yang, G.; Yao, L.; Fu, Q.; Wang, L. Characteristics of Atmospheric Reduced-Sulfur Compounds at a Suburban Site of Shanghai. *Journal of Environmental Sciences* **2025**, *156*, 671–683. https://doi.org/10.1016/j.jes.2024.06.030.
(52) Susaya, J.; Kim, K.-H.; Phan, N.-T.; Kim, J.-C. Assessment of Reduced Sulfur Compounds in Ambient Air as Malodor Components in an Urban Area. *Atmospheric Environment* **2011**, *45* (20), 3381–3390. https://doi.org/10.1016/j.atmosenv.2011.03.051.
(53) Wohl, C.; Forster, G. L.; Edwards, P. M.; Suntharalingam, P.; Oram, D. E. Methanethiol Abundance and Oxidation in a Polluted Marine Atmosphere. *Geophysical Research Letters* **2025**, *52* (8), e2025GL114929. https://doi.org/10.1029/2025GL114929.
(54) Atkinson, R.; Baulch, D. L.; Cox, R. A.; Crowley, J. N.; Hampson, R. F.; Hynes, R. G.; Jenkin, M. E.; Rossi, M. J.; Troe, J. Evaluated Kinetic and Photochemical Data for Atmospheric Chemistry: Volume I - Gas Phase Reactions of O_x, HO_x, NO_x and SO_x

Species. *Atmospheric Chemistry and Physics* **2004**, *4* (6), 1461–1738. https://doi.org/10.5194/acp-4-1461-2004.
